# Self-reported hepatitis B diagnoses and vaccination history in trans and non-binary communities in Germany, the TASG-study* 2022

**DOI:** 10.1186/s12889-026-28677-3

**Published:** 2026-08-11

**Authors:** Renke Biallas, Michael Brandl, Sofia Burdi, Jonas A. Hamm, Chris Spurgat, Kathleen Pöge, Viviane Bremer, Sandra Dudareva, Uwe Koppe

**Affiliations:** 1 Department of Infectious Disease Epidemiology, Robert Koch Institute (RKI), Berlin, Germany; 2 Postgraduate Training for Applied Epidemiology (PAE), Robert Koch Institute (RKI), Berlin, Germany; 3 ECDC Fellowship Programme, Field Epidemiology Path (EPIET), European Centre for Disease Prevention and Control (ECDC), Stockholm, Sweden; 4 Deutsche Aidshilfe (DAH), Berlin, Germany; 5 Department of Epidemiology and Health Monitoring, Robert Koch Institute (RKI), Berlin, Germany; 6 Institute of Public Health, Riga Stradins University, Riga, Latvia

**Keywords:** Epidemiologic studies, Hepatitis B, Sexual and gender minorities, Vaccination

## Abstract

**Background:**

In Germany, little is known about hepatitis B (HepB) prevalence and vaccination coverage among transgender and non-binary people (TNBP), a WHO-defined viral hepatitis key population. Our study aimed to assess self-reported HepB diagnoses, self-reported positive vaccination history, and potential predictors for a positive vaccination history in a subset of participants for whom the German standing committee on vaccination recommend HepB vaccination.

**Methods:**

We analysed data from a 2022 cross-sectional online survey of TNBP in Germany. We calculated percentages and prevalence ratios (PR) with 95% confidence intervals (CI). Predictors of a positive vaccination history among participants potentially recommended for HepB vaccination (e.g. > 1 sexual partner in the last 12 months, people who inject drugs) were identified using a bootstrapped stepwise selection procedure.

**Results:**

We analysed the answers from 2,358 participants. Of these, 0.6% (13/2,358) reported previous HepB diagnosis and the proportion of self-reported positive vaccination history was 56.3% (1,225/2,175). Positive vaccination history varied across subgroups, with higher proportions among participants identifying on the male spectrum than on the female (PR: 1.29; 95% CI: 1.14, 1.47) or non-binary spectrum (PR: 1.27; 95% CI: 1.13, 1.44), participants with higher education levels compared to lower (PR: 1.44, 95% CI: 1.26, 1.68), and in big cities compared to rural areas (PR: 1.22, 95% CI: 1.07, 1.41). Overall, 550 (21.7%) participants were identified as potentially being recommended for HepB vaccination, with a total of 213 participants reporting a negative HepB vaccination history. 65% of these 213 participants were younger than 30 years and should have received the recommended childhood HepB vaccination. The proportion of positive vaccination history increased with the number of hepatitis facts known. The bootstrapped stepwise selection procedure identified knowledge of hepatitis facts as potential predictor for a positive vaccination history (relative selection frequency: 100%).

**Conclusion:**

Self-reported HepB diagnoses were rare among participants and comparable to prevalence estimates in the general population in Germany. To reduce the number of TNBP with unmet vaccination needs and to improve vaccine uptake in this population, gaps in knowledge and lived experiences of TNBP need to be considered in the planning and implementation of targeted interventions. The collection of reliable data is essential on the road to achieve elimination by 2030.

**Supplementary Information:**

The online version contains supplementary material available at 10.1186/s12889-026-28677-3.

## Introduction

Hepatitis B (HepB) is a widespread infectious disease with potentially severe outcomes such as liver cirrhosis and hepatocellular carcinoma [1]. The WHO 2024 Global Hepatitis Report stated that the mortality of viral hepatitis globally increased and defined key populations for focussed prevention efforts, including transgender and non-binary people (TNBP) [2]. A systematic review reported the pooled prevalence of hepatitis B virus infections in transgender populations worldwide to be around 11% (95% CI: 2.0%, 20.0%) [3]. In Germany, the prevalence of active (acute and chronic) HepB infections is estimated to be low in the general population (0.3–0.7%) but may be higher in different subpopulation groups (e.g. people with a migration background, people who inject drugs (PWID)) [4–6]. There is no data systematically collected in Germany on the HepB prevalence among TNBP.

HepB vaccination has been recommended by the German Standing Committee on Vaccination since 1995 for all children and as a standard vaccination for adults with increased risk of sexual exposure including men who have sex with men (MSM), risk of occupational exposure, people frequently receiving blood products, PWID and people living with HIV and/or hepatitis C [7]. While HepB childhood vaccination coverage has reached almost 90% in Germany, coverage in various indication groups (e.g. PWID) is lower or unknown [8–11]. A systematic literature review in 2021 found the hepatitis B vaccine coverage of adults who visited their general practitioner in Germany to be between 22.9–52.1% [9]. Vaccination coverage differed across indication groups, with the highest coverage among children and adolescents (83.0–90.5%) and lower coverage among PWID (10.5–52.5%) and MSM (52.3%) [9]. Data on vaccinations among transgender and non-binary people in Germany is lacking.

TNBP experience many disparities worldwide such as discrimination, violence, and difficulties in accessing prevention and medical services, which result in various health implications [12–15]. Barriers and facilitators of vaccine uptake, particularly against HepB, are under-researched among TNBP but studies for other pathogens exist and provide insights. A review on the uptake of human papilloma virus (HPV) vaccines among LGBTQ individuals found that disease-specific knowledge and diagnosis of any sexually transmitted infections positively influenced the vaccine acceptance [16]. Other barriers such as access to healthcare, emotional barriers (e.g. fear of violence), discrimination and stigmatisation by healthcare providers have been described as factors that influence vaccine hesitancy and the overall vaccination coverage [11, 17, 18].

Using data from the 2022 cross-sectional online survey "Sexual Health and HIV/STI in Trans and Non-Binary Communities" (TASG) [19], we investigated the frequency of self-reported HepB diagnoses, self-reported HepB vaccination history, and knowledge of viral hepatitis facts in TNBP in Germany. We also explored potential predictors of self-reported positive HepB vaccination history among those potentially recommended for HepB vaccination to identify prevention opportunities and unmet needs in this population.

## Methods

### Study design and participants

The TASG study was a participatory cross-sectional study and collaboratively planned, developed, and implemented with representatives of trans and non-binary communities. Between March 1 st and July 1 st 2022, self-identified TNBP aged 18 or older who reside in Germany were invited to participate in an online survey. Access to the online survey was facilitated through various means, including physical distribution (such as cards featuring a QR code leading to the survey website) and electronic dissemination of the survey link through email lists, social media channels, and online banners on websites.

During social media recruitment, we observed an exceptionally high participation from 28.03. to 01.04.2022. Data had therefore to be checked for manipulation attempts. We established data cleaning procedures that have been described elsewhere in more depth [19]. Participants who made negative or offensive comments about TNBP in free text fields, or who did not provide any information other than the obligatory information were excluded. Participants who took part between 28.03. to 01.04.2022 were excluded if they showed a conspicuous response pattern (e.g. always ticking the same answer for different questions). Contradictory or implausible answers were examined and discussed with community representatives.

The TASG study was ethically approved by the Berlin Medical Association (application number: Eth-71/21).

### Measures

For all measures other than the self-reported HepB vaccination history, responses such as “No answer”, or “Don’t know” were coded as missing responses and missing responses were not included in the analysis.

Previously diagnosed HepB was assessed based on the question: "Have you ever been diagnosed with or tested positive for one or more of these sexually transmitted infections (STIs)?” Response answers included multiple sexually transmitted infections (STI) and (1) “Hepatitis B”; (2) “None”; (3) “Don't know”. Responses were dichotomised, categorising those reporting a previous positive HepB test as confirmed previous HepB diagnosis.

Knowledge of hepatitis facts was assessed based on four true statements about viral hepatitis: (1) “Hepatitis is an inflammation of the liver”; (2) “Most hepatitis is caused by viruses”; (3) “There are several types of hepatitis viruses, named after the letters of the alphabet”; (4) “Vaccines exist for both hepatitis A and hepatitis B”. Responses included (1) “I knew that”; (2) “I wasn’t sure about that”; (3) “I didn't know that”; (4) “I don't understand that”; (5) “I don’t believe that’s true”; (6) “No answer”. The responses were dichotomised so that only those who stated that they already knew the fact were categorised as knowing the fact, while all others were not. We also assessed whether respondents knew where to get vaccinated against HepB or not.

The self-reported HepB vaccination history was assessed based on the question: “Have you been vaccinated against hepatitis B?”. Response answers included (1) “No, I’m immune to hepatitis B because I’ve already had it”; (2) “No, and I don’t know if I’m immune or not”; (3) “No, I suffer from chronic hepatitis B”; (4) “Yes, I am fully vaccinated”; (5) “Yes, but I’m not fully vaccinated”; (6) “Yes, but vaccination was unsuccessful”; (7) “Don’t know”; (8) “No answer”. Responses were dichotomised into positive and negative vaccination history, excluding individuals who reported a prior or chronic HepB infection. For a positive vaccination history, answers were categorized based on the answers "Yes, and I completed the series", "Yes, but I did not complete the course", or "Yes, but I did not respond to the vaccinations". A negative vaccination history included the responses "No, and I don't know if I'm immune" or "I don't know".

Using the German HepB vaccination recommendations [7], we considered that anyone reporting more than 1 sexual partner in the past 12 months, current HIV and/or hepatitis C diagnosis, and/or reporting intravenous drug use should be recommended for HepB vaccination.

To further characterise the population and identify prevention potentials and unmet needs, we included in the analysis information on gender identity (“Female spectrum”, “Male spectrum”, “Non-binary female spectrum”, “Non-binary male spectrum”, “Non-binary spectrum”, “Another category”), age (“18–29 years”, “ > = 30 years”), education level (“Low” = up to secondary school diploma, “Medium” = high school diploma, apprenticeship, “High” = university degree, technician or master craftsmen), size of city of residence (“Rural area/village (< = 5,000 inhabitants)”, “City (> 5,000 inhabitants up to < 100,000 inhabitants)”, “Big city (> 100,000 inhabitants)”), monthly income (“no income”, “ < = 2,000€”, “ > 2,000€”), living according to gender identity (“Yes”, “No”, “Partially”), access to health care (“Yes”, “No”), non-utilisation of health services out of fear of being treated inappropriately (“Never”, “Ever in the past years”), frequent discrimination based on gender identity (No (never – sometimes), Yes (usually – always)), severity of depressive symptoms by the Patient Health Questionnaire (PHQ-9) [20, 21] (“No or mild symptoms”, “Moderate symptoms”, “Severe symptoms”), severity of anxiety symptoms by the General Anxiety Disorder questionnaire (GAD-7) [22] (“No or mild symptoms”, “Moderate symptoms”, “Severe symptoms”), internalised trans negativity/positivity (“Rejecting or neutral”, “Agreeing”), body satisfaction (“Yes”, “No”,), previous STI diagnoses (“Yes”, “No”,), receipt of HIV or STI counselling or testing in the last 5 years (“Yes”, “No).

### Data analysis

Descriptive statistics of the participant characteristics include an overview of all main outcomes (self-reported HepB diagnosis, knowledge of hepatitis facts, self-reported HepB vaccination history) and other selected variables. For categorical variables, presentation comprises the total number of respondents and corresponding percentages, with missing values excluded from calculations. For the knowledge variables, we calculated the median number of facts known with the respective interquartile range (IQR) and the proportion of individuals who knew either none, or all four facts. We stratified by self-reported vaccination history (positive vs. negative) for all participants and those potentially recommended for vaccination. We present prevalence ratios and the respective 95% confidence intervals (CI) to allow for comparison across characteristics. Prevalence ratios and CI were estimated by univariate log-binomial regression [23]. We used the bootstrap stepwise selection procedure of Sauerbrei and Schumacher [24] on all variables included in this study to identify potential predictor variables for a positive vaccination history among participants potentially recommended for HepB vaccination. We performed 500 bootstrap replications of the original data set. In each replication, potential predictors were selected by a stepwise backward algorithm that retained only those variables in the logistic regression model that did not exceed our a priori selected cut-off *p*-value of 0.10. The absolute and relative frequency of inclusion was then used as a criterion for the predictive power of each variable. We considered variables as potential predictors if they were selected in the model with a relative selection frequency (RSF) of > 80%.

We ran a sensitivity analysis to assess the impact of excluding the answer “I don’t know” on the overall proportion of participants with a self-reported positive vaccination history. In a second sensitivity analysis we analysed whether demographic characteristics differed between individuals potentially recommended for HepB vaccination and those included in the bootstrapped stepwise selection procedure. Differences were identified visually with a benchmark of 10% deviation indicating a difference in characteristics. In a third sensitivity analysis, when potential predictors had more than two levels, we dichotomized the variable for each level with a constant base level to identify whether any level stood out as a potential predictor.

## Results

**Fig. 1:**
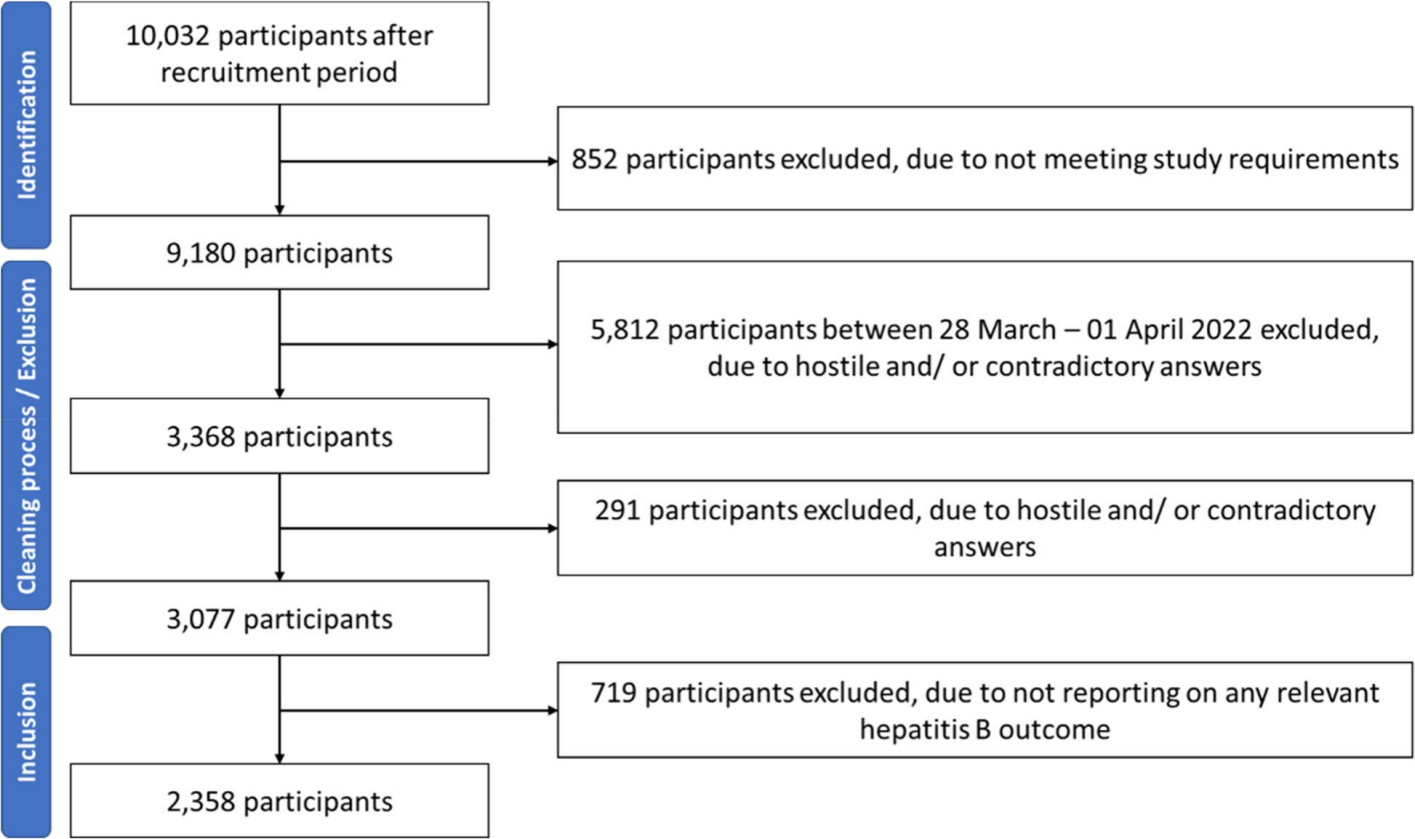
Study participants inclusion flow chart, TASG 2022. Adapted from Pöge K, Spurgat C, Hamm J, et al. [19]

The overall final sample of the TASG study consisted of 3,077 participants. Of the 3,077 participants, 719 (23.4%) did not answer any questions regarding a previous HepB diagnosis, vaccination history or viral hepatitis facts and were excluded from our analyses (Fig. 1). Thus, we analysed data from 2,358 participants.

Characteristics of the analysed study sample are depicted in Table 1. Of the 2,358 included participants, 30.2% identified on the non-binary spectrum, 20.8% on the female spectrum, 22.1% on the male spectrum, 11.8% on the non-binary female spectrum, 14.1% on the non-binary male spectrum, and 1.0% used another category but identified as being on the trans and/or non-binary spectrum. Regarding the age, 62.4% indicated being younger than 30 years (18–29 years). 55.2% indicated to live according to their gender identity in daily life and 38.9% indicated to partially live according to their gender identity. Regarding further demographic characteristics, 15.0% had a low educational level, 61.6% lived in cities with more than 100,000 inhabitants and 80.9% had a monthly income of below 2,000€. Only 20 participants (1.0%) indicated having no access to the German healthcare system.

**Table 1:** Characteristics of participants with no missing responses regarding the hepatitis B outcomes (*n* = 2,358), Germany 2022

	***n* (%)**
Identity category	
Female spectrum	491 (20.8%)
Male spectrum	521 (22.1%)
Non-binary spectrum	712 (30.2%)
Non-binary female spectrum	278 (11.8%)
Non-binary male spectrum	332 (14.1%)
Another category	24 (1.0%)
Age group	
18–29 years	1,472 (62.4%)
> = 30 years	886 (37.6%)
Education level	
Low	297 (15.0%)
Medium	936 (47.2%)
High	748 (37.8%)
Missing	377
City size	
Rural area/village (< = 5.000 inhabitants)	240 (10.4%)
City (> 5.000 inhabitants)	648 (28.1%)
Big city (> 100.000 inhabitants)	1,422 (61.6%)
Missing	48
Monthly income	
No income	121 (6.3%)
< = 2000€	1,437 (74.6%)
> 2000€	369 (19.1%)
Missing	431
Living according to gender identity in daily life	
No	137 (5.9%)
Partially	909 (38.9%)
Yes	1,288 (55.2%)
Missing	24
Number of hepatitis facts known	
0	61 (2.8%)
1	180 (8.2%)
2	485 (22.0%)
3	682 (31.0%)
4	795 (36.1%)
Missing	155

Only 13 of 2,358 participants (0.6%) reported a previous diagnosis of HepB. Of those, seven identified themselves on the female spectrum, three on the non-binary spectrum, one in the male spectrum, one on the non-binary male spectrum, and one identified with another identity category. Of participants with reported previous diagnosis of HepB, nine were 30 years or older and eight indicated living in cities with more than 100,000 inhabitants.

The median number of hepatitis facts participants knew was 3 (IQR: 2–4). Overall, 61 (2.8%) of the respondents who answered the questions regarding the hepatitis facts (*n* = 2,203) did not know of any of the four facts, and 795 (36.1%) knew of all of the four facts (Table 1).

Table 2 shows the self-reported vaccination history across various characteristics. Among the participants who responded to the question regarding HepB vaccination history (*n* = 2,175), 56.3% indicated a positive HepB vaccination history, with comparable distribution across age groups. Regarding gender identity, the proportion of self-reported vaccination history varied across gender identities with the biggest difference between those identifying on the male spectrum and those identifying on the female spectrum (PR: 1.29, 95% CI: 1.14, 1.47).

**Table 2:** Hepatitis B vaccination history of TASG study participants (*n* = 2,175), Germany 2022

	**Negative HepB vaccination history**^a^	**Positive HepB vaccination history**^a^	**PR**^b^	**95% CI**^b^
Overall	950 (43.7%)	1,225 (56.3%)	—	—
Gender identity				
Female spectrum	227 (53.2%)	200 (46.8%)	—	—
Male spectrum	192 (39.4%)	295 (60.6%)	1.29	1.14, 1.47
Non-binary female spectrum	111 (43.2%)	146 (56.8%)	1.21	1.05, 1.40
Non-binary male spectrum	142 (44.4%)	178 (55.6%)	1.19	1.03, 1.37
Non-binary spectrum	270 (40.5%)	397 (59.5%)	1.27	1.13, 1.44
Another category	8 (47.1%)	9 (52.9%)	1.13	0.64, 1.63
Missing	0	0		
Age group				
18–29 years	604 (44.0%)	768 (56.0%)	—	—
> = 30 years	346 (43.1%)	457 (56.9%)	1.02	0.94, 1.10
Missing	0	0		
Education level				
Low	166 (57.0%)	125 (43.0%)	—	—
Medium	406 (43.8%)	521 (56.2%)	1.31	1.14, 1.52
High	282 (38.1%)	459 (61.9%)	1.44	1.26, 1.68
Missing	96	120		
Size of residence city				
Rural area/village (< = 5.000 inhabitants)	118 (51.3%)	112 (48.7%)	—	—
City (> 5.000 inhabitants)	271 (46.1%)	317 (53.9%)	1.11	0.96, 1.30
Big city (> 100.000 inhabitants)	536 (40.8%)	779 (59.2%)	1.22	1.07, 1.41
Missing	25	17		
Monthly income				
No income	58 (49.6%)	59 (50.4%)	—	—
< = 2000€	631 (44.2%)	795 (55.8%)	1.11	0.93, 1.35
> 2000€	139 (38.1%)	226 (61.9%)	1.23	1.02, 1.52
Missing	122	145		
Living according to gender identity in daily life				
No	58 (53.7%)	50 (46.3%)	—	—
Partially	363 (43.2%)	478 (56.8%)	1.23	1.01, 1.54
Yes	521 (43.0%)	692 (57.0%)	1.23	1.02, 1.55
Missing	8	5		
Number of hepatitis facts known				
0	50 (90.9%)	5 (9.1%)	—	—
1	143 (82.2%)	31 (17.8%)	1.96	0.88, 5.53
2	253 (52.8%)	226 (47.2%)	5.19	2.53, 14.1
3	283 (41.9%)	393 (58.1%)	6.39	3.13, 17.3
4	216 (27.6%)	566 (72.4%)	7.96	3.90, 21.6
Missing	5	4		

^a^*n* (row %)

^b^PR = Prevalence ratio

*CI* Confidence Interval

Higher proportions of self-reported positive HepB vaccination history were found in participants with higher levels of education (PR: 1.44, 95% CI: 1.26, 1.68), living in big cities (PR: 1.22, 95% CI: 1.07, 1.41), living partially or always according to gender identity (PR: 1.23, 95% CI: 1.01, 1.54; PR: 1.23, 95% CI: 1.02, 1.55), and knowing four hepatitis facts compared to those who knew zero facts (PR: 7.96, 95% CI: 3.90, 21.6).

We identified 550 participants (21.7%) for whom HepB vaccination was potentially recommended based on available variables**.** Of those, 61.3% reported a positive HepB vaccination history (Table 3). We found that respondents identifying on the male spectrum reported more frequently a positive vaccination history than other respondents (Table 3). As in the overall sample, we found positive vaccination history more often among those with higher education (PR: 1.37, 95% CI: 1.06, 1.89) and in participants living partially (PR: 1.78, 95% CI: 1.11, 3.53) or always (PR: 1.80, 95% CI: 1.13, 3.57) according to their gender identity. Regarding healthcare and prevention-related factors we found more respondents with positive HepB vaccination history among those who received HIV or STI counselling or testing in the past 5 years than those who did not (PR: 1.34, 95% CI: 1.17, 1.54). Positive vaccination history was more often reported among participants with increasing level of hepatitis knowledge, with the biggest difference in those who knew of all four facts compared to those who knew zero facts (PR: 4.34, 95% CI: 1.93, 16.30). The first sensitivity analysis showed that the proportion of self-reported positive HepB vaccination history increased drastically when excluding the answer “I don’t know” in the analysis, from 56.3% to 79.9% (1.225/1.534). Among participants younger than 30 years of age the proportion increased from 56.0 to 83.6% (768/919).

**Table 3:** Vaccination history of TASG study participants for whom HepB vaccination is recommended (*n* = 550), Germany 2022

	Negative HepB vaccination history^a^	Positive HepB vaccination history^a^	PR^b^	95% CI^c^
Overall	213 (38.7%)	337 (61.3%)	—	—
**Gender identity**				
Female spectrum	54 (48.6%)	57 (51.4%)	—	—
Male spectrum	30 (31.9%)	64 (68.1%)	1.32	1.05, 1.68
Non-binary female spectrum	32 (42.7%)	43 (57.3%)	1.13	0.86, 1.47
Non-binary male spectrum	30 (38.0%)	49 (62.0%)	1.22	0.94, 1.57
Non-binary spectrum	64 (34.4%)	122 (65.6%)	1.29	1.05, 1.61
Another category	3 (60.0%)	2 (40.0%)	0.65	0.13, 1.46
Missing	0	0		
**Age group**				
18–29 years	139 (40.8%)	202 (59.2%)	—	—
> = 30 years	74 (35.4%)	135 (64.6%)	1.09	0.95, 1.25
Missing	0	0		
**Education level**				
Low	27 (50.0%)	27 (50.0%)	—	—
Medium	81 (38.2%)	131 (61.8%)	1.28	0.98, 1.77
High	73 (34.0%)	142 (66.0%)	1.37	1.06, 1.89
Missing	32	37		
**Size of residence city**				
Rural area/village (< = 5.000 inhabitants)	16 (43.2%)	21 (56.8%)	—	—
City (> 5.000 inhabitants)	43 (41.7%)	60 (58.3%)	1.01	0.74, 1.45
Big city (> 100.000 inhabitants)	144 (36.5%)	251 (63.5%)	1.12	0.87, 1.57
Missing	10	5		
**Monthly income**				
No income	11 (55.0%)	9 (45.0%)	—	—
< = 2000€	133 (37.5%)	222 (62.5%)	1.46	0.97, 2.68
> 2000€	31 (32.6%)	64 (67.4%)	1.57	1.02, 2.91
Missing	38	42		
**Living according to gender identity in daily life**				
No	14 (63.6%)	8 (36.4%)	—	—
Partially	78 (37.5%)	130 (62.5%)	1.78	1.11, 3.53
Yes	118 (37.2%)	199 (62.8%)	1.80	1.13, 3.57
Missing	3	0		
**Body satisfaction**				
No	67 (33.3%)	134 (66.7%)	—	—
Yes	138 (41.1%)	198 (58.9%)	0.88	0.77, 1.01
Missing	8	5		
**Frequent discrimination based on gender identity?**				
No (never – sometimes)	145 (38.3%)	234 (61.7%)	—	—
Yes (usually – always)	43 (39.4%)	66 (60.6%)	0.98	0.82, 1.16
Missing	25	37		
**Position to statements of internalized trans negativity**				
Rejecting or neutral	159 (37.0%)	271 (63.0%)	—	—
Agreeing	44 (47.3%)	49 (52.7%)	0.83	0.66, 1.00
Missing				
**Position to statements of internalized trans positivity**				
Rejecting or neutral	116 (40.6%)	170 (59.4%)	—	—
Agreeing	88 (36.7%)	152 (63.3%)	1.06	0.92, 1.21
Missing	9	15		
**Patient Health Questionnaire (PHQ-9)**				
No or mild symptoms	84 (37.8%)	138 (62.2%)	—	—
Moderate symptoms	52 (32.5%)	108 (67.5%)	1.08	0.93, 1.26
Severe symptoms	70 (46.1%)	82 (53.9%)	0.87	0.72, 1.03
Missing	7	9		
**Generalized Anxiety Disorder 7-item (GAD-7)**				
No or mild symptoms	114 (37.0%)	194 (63.0%)	—	—
Moderate symptoms	59 (40.1%)	88 (59.9%)	0.96	0.81, 1.11
Severe symptoms	32 (39.5%)	49 (60.5%)	0.95	0.77, 1.15
Missing	8	6		
**Non-utilisation of health services out of fear of being treated inappropriately?**				
Never—more than 5 years ago	39 (45.3%)	47 (54.7%)	—	—
Within the past 5 years	120 (34.8%)	225 (65.2%)	1.19	0.99, 1.50
Missing	54	65		
**Received any HIV or STI counselling or testing in the past 5 years?**				
No	129 (47.3%)	144 (52.7%)	—	—
Yes	84 (30.3%)	193 (69.7%)	1.34	1.17, 1.54
Missing	0	0		
**At least one STI diagnosis ever**				
No	168 (39.5%)	257 (60.5%)	—	—
Yes	43 (36.8%)	74 (63.2%)	0.93	0.71, 1.21
Missing	2	6		
**Number of hepatitis facts known**				
0	13 (81.3%)	3 (18.7%)	—	—
1	36 (85.7%)	6 (14.3%)	0.77	0.23, 3.34
2	62 (50.4%)	61 (49.6%)	2.81	1.22, 10.60
3	50 (33.3%)	100 (66.7%)	3.78	1.67, 14.20
4	51 (23.4%)	167 (76.6%)	4.34	1.93, 16.30
Missing	1	0		
**Knowledge of where to get vaccinated**				
No	131 (89.7%)	15 (10.3%)	—	—
Yes	80 (81.6%)	18 (18.4%)	1.81	0.96, 3.48
Missing	2	304		

^a^*n* (row %)

^b^PR = Prevalence Ratio

^c^CI = Confidence Interval

To identify potential predictors for self-reported positive HepB vaccination history among participants potentially recommended for HepB vaccination, the bootstrap replication process showed that in a subsample with no missing responses (*n* = 337), knowledge of hepatitis facts (number of facts known) was a potential predictor for a positive vaccination history (Table 4). Other factors were below the defined threshold (< 80%). Knowledge regarding places to get vaccinated was excluded from this analysis due to a low number of responses (246/550, 44.7%). The second sensitivity analysis showed that all difference in demographic variables were within 10% with the biggest difference below 5%, indicating no apparent deviation (Additional file 1). The third sensitivity analysis, using dichotomized knowledge levels, showed that knowing 3 or 4 facts compared to 0 facts were identified as potential predictors for a positive HepB vaccination history among participants potentially recommended for HepB vaccination (Additional file 2).

**Table 4:** Absolute and relative bootstrap replication inclusion frequencies of potential predictors for positive HepB vaccination history among participants potentially recommended for HepB vaccination (*n* = 337)

Variable	Absolute frequencies	Relative frequencies*
Knowledge of hepatitis facts	500	100.0%
Receipt of counselling or testing in the past 5 years	348	69.6%
Avoidance of medical service in the past 5 years	304	60.8%
Size of residence city	195	39.0%
Living according to gender identity	122	24.4%
Gender identity	109	21.8%
Body satisfaction	92	18.4%
Education level	88	17.6%
Patient Health Questionnaire (PHQ-9)	86	17.2%
Generalized Anxiety Disorder 7-item (GAD-7)	81	16.2%
Internalised trans negativity	67	13.4%
Monthly income	61	12.2%
Age group	60	12.0%
Internalised trans positivity	44	8.8%
Frequent discrimination based on gender identity	44	8.8%
At least one previous STI diagnosis	29	5.8%

^*^values with a relative frequency of > = 80% were considered prognostically meaningful

**Fig. 2:**
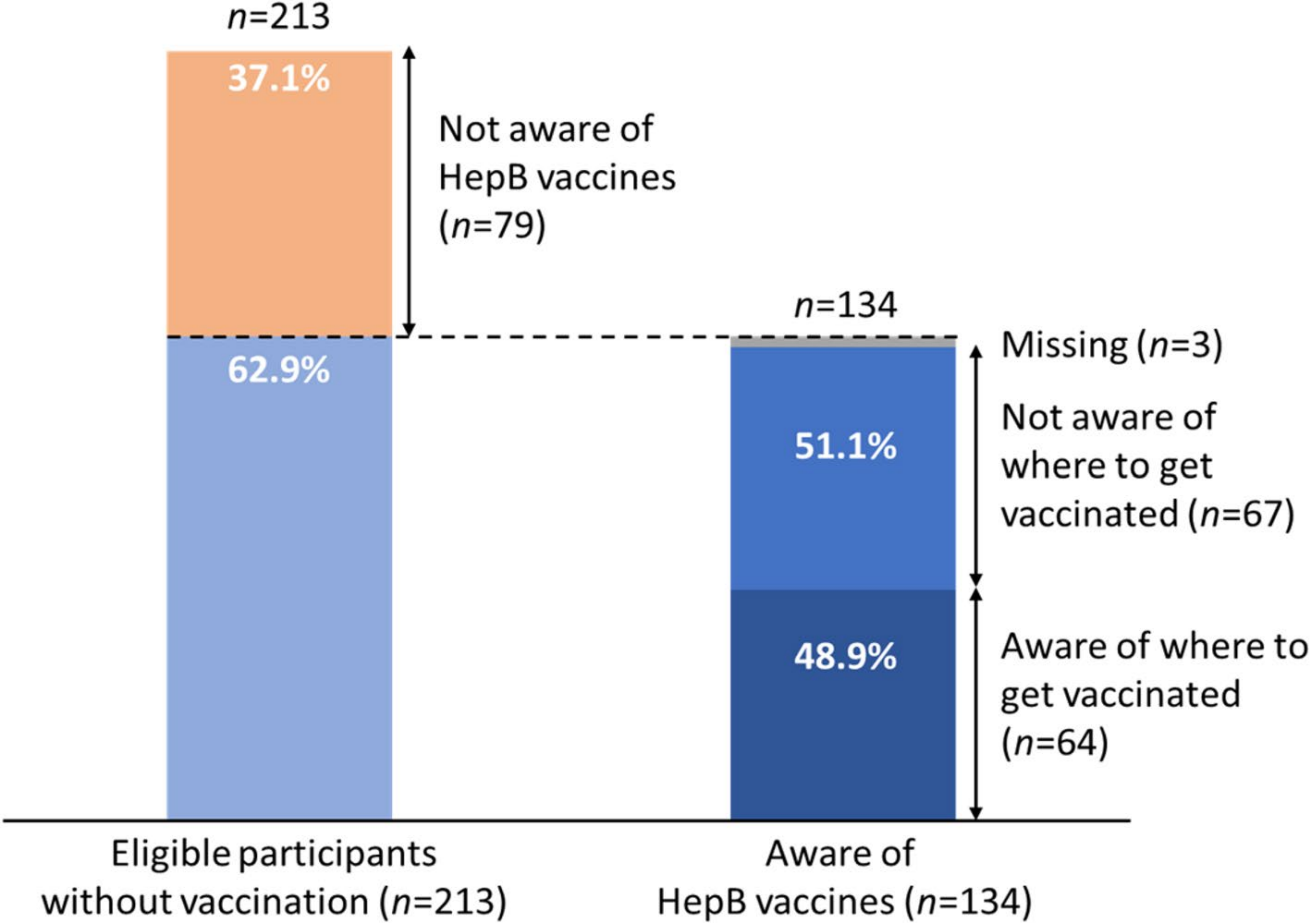
Knowledge regarding Hepatitis B (HepB) vaccination among participants potentially recommended for HepB vaccination of the TASG study (*n* = 213), Germany 2022

In total, 213 participants potentially recommended for HepB vaccination reported a negative vaccination history. Of these 213 participants, 65.3% (*n* = 139) were aged younger than 30 years and should have been vaccinated against HepB during childhood. Overall, 79 (37.1%) of these participants did not know about available vaccines (Fig. 2). Of the 131 participants who knew of the vaccines, 67 (51.1%) did not know where to get vaccinated. Of all participants potentially recommended for vaccination with negative HepB vaccination history, 64/213 (30.0%) knew that HepB vaccines are available and where to get vaccinated.

## Discussion

In this manuscript we describe the prevalence of self-reported previous HepB diagnoses and the proportion of self-reported positive vaccination history among TNBP in Germany. In the overall study sample self-reported HepB diagnoses were rare (0.6%) and the self-reported positive HepB vaccination history low (56.3%). Among participants potentially recommended for HepB vaccination, the proportion of positive HepB vaccination history was also low (61.3%) and efforts to increase vaccination uptake are needed. Knowledge of hepatitis facts was identified as a potential predictor of positive vaccination history.

The low prevalence of self-reported HepB diagnoses in the study population aligns with the low prevalence estimates of HepB in the general population of Germany that are based on serologically confirmed data (0.3–1.6%) [5, 6]. As not all HepB infections are diagnosed, with the majority of infections being asymptomatic, the presented proportion of people reporting HepB diagnosis does not reflect the overall prevalence of hepatitis B virus infections but rather the prevalence of diagnosed infections. Nevertheless, our findings contrast with a systematic review conducted in 2022 which reported a pooled global prevalence of 11.0% (95% CI: 2.0%, 20.0%) in transgender people and a prevalence in the United States of 16.0% (95% CI: 8.0%, 23.0%) [3]. The authors of the systematic review found three studies on the prevalence of HepB among trans female persons and reported a combined prevalence of HepB of 6.0% (95% CI: 3.0%, 10.0%) [3]. Differences from other study results are expected due to differing study designs (i.e. seroprevalence studies), sampling strategies, recruited population, and health systems. Some but not all of the studies (conducted in the US) included in the systematic review focussed on TNBP that are part of other key populations such as sex workers, incarcerated people, PWID, people living with HIV. In our sample generated through online recruitment, we might not have reached as many individuals at higher risk for HepB (e.g. being marginalised in multiple ways) [3]. The prevalence of self-reported HepB diagnoses could be higher within these subpopulations in Germany.

The proportion of self-reported positive HepB vaccination history of 56.3% in the study sample is higher than the overall estimated vaccination coverage in the adult population in Germany (22.9–52.1%) but could nevertheless be improved [9]. HepB vaccination coverage in Germany has been described to vary across different populations, with lower vaccination coverage among selected key populations such as PWID (10.5–52.5%) [9]. Another more recent study focusing on MSM in Germany, a population group with active vaccination recommendation, found a HepB vaccination coverage of approximately 80% [25]. Data on vaccination coverage among TNBP is not collected systematically in Germany. The presented results may have been biased as regularly collected and published data on recommended childhood vaccinations in Germany by the federal health monitoring system, shows that already in 2004, 84% of children were vaccinated against HepB at school entry [26]. This proportion continued for the following years to be as high or higher, indicating a relatively high vaccination coverage in the population aged under 30 years in 2022 [10]. Nevertheless, the proportion of participants reporting a positive HepB vaccination history in our study sample did not differ regarding age. The overall proportion of self-reported positive vaccination history of 56.0% among those aged < 30 years is likely an underestimation, because participants who did not know their vaccination history were grouped as not vaccinated (comparison with first sensitivity analysis). Nevertheless, TNBP are considered a key population for hepatitis prevention and should therefore have regular access to screening for infection and immunisation status. A self-reported negative HepB vaccination history therefore also implies potential unmet prevention needs or gaps in medical check-ups. This applies especially to younger participants (62.4% of the overall study population) who should have received a recommended HepB childhood vaccination but did not recall this event correctly. As the proportion of self-reported positive HepB vaccination history in the overall sample differed across gender identities, city size and education, future interventions should aim to improve vaccination uptake among all TNBP with a special focus on TNBP identifying on the female spectrum, those living in rural areas and those with lower education.

Within our sample we identified a subgroup of TNBP, that is potentially at risk for exposure to the HepB virus and therefore based on available TASG variables recommended for vaccination. The proportion of self-reported positive HepB vaccination history was still low (61.3%) in this subgroup, especially considering that all participants within this subgroup potentially fall under the German vaccination recommendations. The number of hepatitis facts known was identified as a potential predictor of positive HepB vaccination history within this subgroup. The proportion of self-reported positive vaccination history in this subgroup was higher among those who received HIV or STI counselling or testing in the past 5 years. This is consistent with findings from a study on HepB vaccination among MSM in Germany, which reported an association between disease-specific knowledge and positive vaccination history and highlighted the importance of awareness of available vaccines and vaccination sites for informed decision-making [25]. However, due to the cross-sectional design of our study, causal relationships between hepatitis-related knowledge and self-reported positive vaccination history cannot be inferred as the temporal sequence between the two remains unclear. Participants with lower levels of knowledge may also be less aware of their vaccination status, which could have led to underestimation of self-reported positive vaccination history. Even though within our sample, no other variables were identified as potential predictors of self-reported positive HepB vaccination history, multiple barriers for transgender people have been described in the literature. Potentially relevant barriers include fear of discrimination, misgendering by the health provider, and lack of access to health systems [18, 27, 28]. In our study sample, some participants recommended for HepB vaccination were unaware of available HepB vaccines or did not know where to get vaccinated. Many knew of the vaccines and where to get vaccinated but did not report any previous HepB vaccination. Lack of knowledge of vaccines has previously been described as a major cause for vaccine hesitancy [29]. Other reasons for vaccine hesitancy discussed in the literature, include geographic barriers and disease risk perception [29]. A sound understanding of reasons for non-vaccination and vaccine hesitancy is essential to develop tailored campaigns and education programmes to improve vaccine uptake in this specific demographic group [29–31]. As the proportion of self-reported positive HepB vaccination history in the subgroup was higher among those with increased levels of hepatitis knowledge and those who received testing or counselling, future interventions to improve vaccine uptake should aim to increase awareness of available testing and counselling and to improve knowledge about viral hepatitis and associated risk factors among TNBP at risk for HepB. Not all TNBP regularly use the available HIV or STI testing and counselling. General practitioners and other institutions that offer testing for the general population should be aware of the HepB vaccination recommendations for individuals at risk for infection and also be aware of the needs of key populations. The qualitative project part of the TASG project has shown two facts about the available testing and counselling offers that were not specifically targeted towards TNBP: (1) they are not seen as meeting the needs of TNBP; (2) they were not perceived as safe and free of discrimination. Hence, TNBP often avoided these facilities even when they needed the services [19]. It is essential to provide services perceived as competent and safe by TNBP and use targeted information campaigns to disseminate the information of existing offers within these communities. The implementation of universal childhood vaccination programs may also help to achieve a high vaccination coverage and decrease of new infections over time. However, a modelling study from the Netherlands showed that solely relying on general vaccination policies is not sufficient in the end [32]. This further supports the continued need for targeted interventions to improve vaccine uptake in key populations.

Some limitations should be considered while interpreting the results. The methodological reliance on self-reporting may introduce recall bias (especially for vaccination history), and the cross-sectional design of the study limits our ability to establish causal relationships. Evidence from Switzerland suggests that self-reported indicators as vaccination history likely overestimate vaccination coverage but perform well in detecting individuals with previous vaccination [33]. Assessing the self-reported vaccination history therefore still gives valuable insights. Other relevant variables that need to be considered regarding HepB vaccination include profession, travel history and migration history that are covered by the standing vaccination recommendations. Thus, the proportion of participants recommended for HepB vaccination in our study might be an underestimate. The unmet needs of these people might differ from the described participants. The reliance on a convenience sample may have introduced a selection bias as more vulnerable individuals that are marginalised in many ways (part of multiple marginalised groups at the same time) might not have been reached. The categorisation of eligibility for the HepB vaccination was based on available responses. This categorisation therefore does not consider any further contextual factors, explicitly that having multiple HepB-vaccinated sexual partners, does not necessarily reflect a higher risk for the disease. We did not include laboratory methods to confirm previous or recent infections nor serology in this study, but this could be considered in future research proposals (e.g. seroprevalence studies). While our findings seem to align with serological national estimates, the prevalence is likely underestimated within our sample due to high proportions of asymptomatic HepB infections. With the data and the relatively small number of participants recommended for vaccination, we could not identify further factors or barriers that potentially affect the vaccine uptake. As mentioned in the method section, we encountered a high number of trans-hostile commentators and calls for fake participation during the recruiting phase, aimed at manipulating the study. This resulted in immense data cleaning, excluding a high number of responses from the dataset. Nevertheless, it is still possible that some of the hostile commentors remained in the dataset while eligible participants were excluded.

Despite these limitations, the TASG study gives insights into the health of transgender and non-binary communities in Germany. We were able to recruit a large study sample of TNBP in Germany and analyse responses to describe relevant health outcomes such as self-reported HepB diagnosis and self-reported HepB vaccination history for the first time in Germany and identify prevention opportunities. Involving representatives of transgender and non-binary communities ensured the appropriateness of the design of the study, questionnaire and analyses.

## Conclusion

Our study provides insights into HepB diagnoses, vaccination history, and hepatitis knowledge among TNBP in Germany. While previous HepB diagnoses were rarely reported, our study found unmet prevention needs based on the self-reported positive HepB vaccination history among participants potentially recommended for HepB vaccination. To further enhance vaccine uptake and promote the overall health of TNBP, it is important to address knowledge gaps and consider individual lived experiences of persons with trans and non-binary identities and educational levels. Our findings provide a foundation for further research and collaborative efforts to enhance the health and well-being of trans and non-binary individuals in Germany. Tailored interventions like counselling offers that consider the lived experiences and diverse needs of trans and non-binary communities as well as the collection of reliable data are essential on the path towards elimination of HepB.

## Supplementary Information

Additional file 1.

Additional file 2.

Additional file 3.

## Data Availability

The datasets used and/or analysed during the current study are available from the corresponding author on reasonable request.

## References

[1] World Health Organization. Hepatitis B: key facts [ https://www.who.int/news-room/fact-sheets/detail/hepatitis-b]

[2] Global hepatitis report. action for access in low- and middle-income countries. In. Geneva: World Health Organization; 2024. p. 2024.

[3] Moradi G, Soheili M, Rashti R, Dehghanbanadaki H, Nouri E, Zakaryaei F, et al. The prevalence of hepatitis C and hepatitis B in lesbian, gay, bisexual and transgender populations: a systematic review and meta-analysis. Eur J Med Res. 2022;27(1):47. 35346371 10.1186/s40001-022-00677-0 PMC8962539

[4] Dudareva S, Faber M, Zimmermann R, Bock CT, Offergeld R, Steffen G, et al. Epidemiology of viral hepatitis A to E in Germany. Bundesgesundheitsbl Gesundheitsforsch Gesundheitsschutz. 2022;65(2):149–58. 10.1007/s00103-021-03478-8 PMC8758919 35029725

[5] Poethko-Müller C, Zimmermann R, Hamouda O, Faber M, Stark K, Ross RS, et al. Epidemiology of hepatitis A, B, and C among adults in Germany: results of the German Health Interview and Examination Survey for Adults (DEGS1). Bundesgesundheitsbl Gesundheitsforsch Gesundheitsschutz. 2013;56(5–6):707–15. 10.1007/s00103-013-1673-x 23703489

[6] Sperle I, Steffen G, Leendertz SA, Sarma N, Beermann S, Thamm R, Simeonova Y, Cornberg M, Wedemeyer H, Bremer V et al: Prevalence of Hepatitis B, C, and D in Germany: results from a scoping review. Front Public Health 2020;8. 10.3389/fpubh.2020.00424 PMC7493659 33014960

[7] Ständige Impfkommission. Empfehlungen der Ständigen Impfkommission (STIKO) beim Robert Koch-Institut 2023. In: Epid bull, vol. vol. 4. Epid Bull: Robert Koch-Institut; 2023. p. 3–68.

[8] Brandl M, Schmidt AJ, Marcus U, An der Heiden M, Dudareva S. Are men who have sex with men in Europe protected from hepatitis B? Epidemiol Infect. 2020;148:e27. 32052715 10.1017/S0950268820000163 PMC7026898

[9] Steffen G, Sperle I, Harder T, Sarma N, Beermann S, Thamm R, et al. Hepatitis B vaccination coverage in Germany: systematic review. BMC Infect Dis. 2021;21:1–17. 34391406 10.1186/s12879-021-06400-4 PMC8364709

[10] Rieck T, Feig M, Siedler A. Impfquoten von Kinderschutzimpfungen in Deutschland – aktuelle Ergebnisse aus der RKI-Impfsurveillance. Epidemiol Bull. 2022;48:3–25.

[11] Brandl M, Schmidt AJ, Marcus U, Duffell E, Severi E, Mozalevskis A, Kivite-Urtane A, an der Heiden M, Dudareva S: Self-reported hepatitis A and B vaccination coverage among men who have sex with men (MSM), associated factors, and vaccination recommendations in 43 countries of the WHO European Region: results from the European MSM Internet Survey, EMIS-2017. Euro Surveill. 2024;29(45):2400100. 10.2807/1560-7917. 10.2807/1560-7917.ES.2024.29.45.2400100 PMC11544724 39512170

[12] James SE, Herman JL, Rankin S, Keisling M, Mottet L, Anafi M. The report of the 2015 U.S. transgender survey. Washington DC: National Center for Transgender Equality; 2016.

[13] McCann E, Donohue G, Brown M. Experiences and perceptions of trans and gender non-binary people regarding their psychosocial support needs: a systematic review of the qualitative research evidence. Int J Environ Res Public Health. 2021;18(7):3403. 33806008 10.3390/ijerph18073403 PMC8036290

[14] Skuban-Eiseler T, Orzechowski M, Steger F. Why do transgender individuals experience discrimination in healthcare and thereby limited access to healthcare? An interview study exploring the perspective of German transgender individuals. Int J Equity Health. 2023;22(1):211. 37817187 10.1186/s12939-023-02023-0 PMC10566060

[15] Warner DM, Mehta AH. Identifying and addressing barriers to transgender healthcare: where we are and what we need to do about it. J Gen Intern Med. 2021;36(11):3559–61. 34254221 10.1007/s11606-021-07001-2 PMC8606364

[16] Hao Z, Guo Y, Bowling J, Ledenyi M. Facilitators and barriers of HPV vaccine acceptance, initiation, and completion among LGBTQ community in the U.S.: a systematic review. Int J Sex Health. 2022;34(2):291–307. 38596525 10.1080/19317611.2021.1989535 PMC10903696

[17] Azucar D, Slay L, Valerio DG, Kipke MD. Barriers to COVID-19 vaccine uptake in the LGBTQIA community. Am J Public Health. 2022;112(3):405–7. 35196061 10.2105/AJPH.2021.306599 PMC8887181

[18] Balaji JN, Prakash S, Joshi A, Surapaneni KM. A scoping review on COVID-19 vaccine hesitancy among the lesbian, gay, bisexual, transgender, queer, intersex and asexual (LGBTQIA+) community and factors fostering its refusal. Healthcare (Basel). 2023;11(2):245. 36673613 10.3390/healthcare11020245 PMC9859126

[19] Pöge K, Spurgat C, Hamm J, Koppe U: Forschungsbericht zum Projekt “Sexuelle Gesundheit und HIV/STI in trans und nicht-binären Communitys”. 2023. [last accessed: 27.07.2026]https://www.rki.de/DE/Themen/Infektionskrankheiten/Infektionskrankheiten-A-Z/H/HIV-AIDS/Studien/TASG-Ergebnisse.html?nn=16911154

[20] Gräfe K, Zipfel S, Herzog W, Löwe B. Screening psychischer Störungen mit dem “Gesundheitsfragebogen für Patienten (PHQ-D)“. Diagnostica (Bologna). 2004;50(4):171–81.

[21] Kroenke K, Spitzer RL, Williams JB. The PHQ-9: validity of a brief depression severity measure. J Gen Intern Med. 2001;16(9):606–13. 11556941 10.1046/j.1525-1497.2001.016009606.x PMC1495268

[22] Löwe B, Decker O, Müller S et al. Validation and standardization of the Generalized Anxiety Disorder Screener (GAD-7) in the general population. Medical care. 2008;46:266–274. 10.1097/MLR.0b013e318160d093. 10.1097/MLR.0b013e318160d093 18388841

[23] Log-binomial regression to estimate a risk ratio or prevalence ratio [https://www.bookdown.org/rwnahhas/RMPH/]

[24] Sauerbrei W, Schumacher M. A bootstrap resampling procedure for model building: application to the cox regression model. Stat Med. 1992;11(16):2093–109. 1293671 10.1002/sim.4780111607

[25] Esber AL, Streeck H, Jansen K, Dorsey-Spitz J, Robb ML, Crowell TA. High hepatitis B vaccination coverage among a cohort of predominantly men who have sex with men in Germany. Hum Vaccin Immunother. 2023;19(2):2238576. 37489272 10.1080/21645515.2023.2238576 PMC10392733

[26] Gesundheitsberichterstattung des Bundes [] ; last accessed: 23.07.2026 https://www.gbe-bund.de

[27] Kcomt L, Gorey KM, Barrett BJ, McCabe SE. Healthcare avoidance due to anticipated discrimination among transgender people: a call to create trans-affirmative environments. SSM Popul Health. 2020;11:100608. 32529022 10.1016/j.ssmph.2020.100608 PMC7276492

[28] Thomas SD, King R, Murphy M, Dempsey M. Demographic factors associated with healthcare avoidance and delay in the transgender population: findings from a systematic review. Dialogues Health. 2023;3:100159. 38515802 10.1016/j.dialog.2023.100159 PMC10954025

[29] Marti M, de Cola M, MacDonald NE, Dumolard L, Duclos P. Assessments of global drivers of vaccine hesitancy in 2014—looking beyond safety concerns. PLoS ONE. 2014;12(3):e0172310. 10.1371/journal.pone.0172310 PMC5332020 28249006

[30] Habersaat KB, Jackson C. Understanding vaccine acceptance and demand—and ways to increase them. Bundesgesundheitsbl Gesundheitsforsch Gesundheitsschutz. 2020;63(1):32–9. 10.1007/s00103-019-03063-0 PMC6925076 31802154

[31] Tuckerman J, Kaufman J, Danchin M. Effective approaches to combat vaccine hesitancy. Pediatr Infect Dis J. 2022;41(5):e243–5. 35213864 10.1097/INF.0000000000003499 PMC8997018

[32] Xiridou M, Visser M, Urbanus A, Matser A, van Benthem B, Veldhuijzen I. Ending risk-group HBV vaccination for MSM after the introduction of universal infant HBV vaccination: a mathematical modelling study. Vaccine. 2021;39(21):2867–75. 33896665 10.1016/j.vaccine.2021.04.008

[33] Schmidt AJ, Rasi M, Esson C, Christinet V, Ritzler M, Lung T, et al. The Swiss STAR trial - an evaluation of target groups for sexually transmitted infection screening in the sub-sample of men. Swiss Med Wkly. 2020;150:w20392. 33382077 10.4414/smw.2020.20392

