# Supplementary material for: Self-reported hepatitis B diagnoses and vaccination history in trans and non-binary communities in Germany, the TASG-study* 2022
Source: BMC Public Health. 2026 Aug 11;26:2321. doi: 10.1186/s12889-026-28677-3 (PMC13460023; doi:10.1186/s12889-026-28677-3)
Supplement: Supplementary file 1 [file 12889_2026_28677_MOESM1_ESM.docx]

**Annex 1.** Comparison of characteristics of participants potentially recommended for HepB vaccination and participants included in the bootstrapped stepwise selection model (*n*=3,077), Germany 2022.

| **Characteristic** | **Potentially recommended for HepB vaccination,**  *N* = 550^1^ | **Included in bootstrapped stepwise selection model**,  *N* = 337 |
| --- | --- | --- |
| **Identity category** |  |  |
| Female spectrum | 111 (20%) | 66 (20%) |
| Male spectrum | 5 (0.9%) | 4 (1.2%) |
| Non-binary | 94 (17%) | 69 (20%) |
| Non-binary female spectrum | 186 (34%) | 105 (31%) |
| Non-binary male spectrum | 75 (14%) | 42 (12%) |
| Another category | 79 (14%) | 51 (15%) |
| **Age group** |  |  |
| 18–29 years | 341 (62%) | 197 (58%) |
| >= 30 years | 209 (38%) | 140 (42%) |
| **Education level** |  |  |
| Low | 54 (11%) | 30 (8.9%) |
| Medium | 212 (44%) | 151 (45%) |
| High | 215 (45%) | 156 (46%) |
| **City size** |  |  |
| Rural area/village (<= 5.000 inhabitants) | 37 (6.9%) | 19 (5.6%) |
| City (> 5 000 inhabitants) | 103 (19%) | 62 (18%) |
| Big city (> 100 000 inhabitants) | 395 (74%) | 256 (76%) |
| **Monthly income** |  |  |
| No income | 20 (4.3%) | 12 (3.6%) |
| <= 2000€ | 355 (76%) | 252 (75%) |
| >2000€ | 95 (20%) | 73 (22%) |
| ^1^*n* (row %) |  |  |
