# Supplementary material for: Self-reported hepatitis B diagnoses and vaccination history in trans and non-binary communities in Germany, the TASG-study* 2022
Source: BMC Public Health. 2026 Aug 11;26:2321. doi: 10.1186/s12889-026-28677-3 (PMC13460023; doi:10.1186/s12889-026-28677-3)
Supplement: Supplementary file 2 [file 12889_2026_28677_MOESM2_ESM.docx]

**Annex 2.** Absolute and relative bootstrap replication inclusion frequencies of potential predictors for positive HepB vaccination history among participants recommended for HepB vaccination (*n*=337).

| **Variable** | **Absolute frequencies** | **Relative frequencies*** |
| --- | --- | --- |
| Knowledge of 4 hepatitis facts | 500 | 100,0% |
| Knowledge of 3 hepatitis facts | 500 | 100,0% |
| Avoidance of medical services in the past 5 years | 177 | 35.4% |
| Frequent discrimination based on gender identity | 163 | 32.6% |
| Size of residence city | 149 | 29.8% |
| Generalized Anxiety Disorder 7-item (GAD-7) | 139 | 27.8% |
| Body satisfaction | 133 | 26.6% |
| Living according to gender identity | 126 | 25.2% |
| Patient Health Questionnaire (PHQ-9) | 121 | 24.2% |
| Internalised trans negativity | 120 | 24.0% |
| Received counselling or testing in the past 5 years | 93 | 18.6% |
| Age group | 81 | 16.2% |
| Knowledge of 2 hepatitis facts | 78 | 15.6% |
| Internalised trans positivity | 74 | 14.8% |
| Education level | 66 | 13.2% |
| At least one previous STI diagnosis | 56 | 11.2% |
| Gender identity | 53 | 10.6% |
| Monthly income | 47 | 9.4% |
| Knowledge of 1 hepatitis fact | 7 | 1.4% |
| *values with a relative frequency of >=80% were considered prognostically meaningful | | |
