# Supplementary material for: Self-reported hepatitis B diagnoses and vaccination history in trans and non-binary communities in Germany, the TASG-study* 2022
Source: BMC Public Health. 2026 Aug 11;26:2321. doi: 10.1186/s12889-026-28677-3 (PMC13460023; doi:10.1186/s12889-026-28677-3)
Supplement: Supplementary file 3 [file 12889_2026_28677_MOESM3_ESM.docx]

STROBE Statement—checklist of items that should be included in reports of observational studies

|  | | Item No. | Recommendation | | Page  No. | | | Relevant text from manuscript | |
| --- | --- | --- | --- | --- | --- | --- | --- | --- | --- |
| **Title and abstract** | | 1 | (*a*) Indicate the study’s design with a commonly used term in the title or the abstract | | 1 | | | We analysed data from a 2022 cross-sectional online survey of TNBP in Germany. | |
|  |  |  | (*b*) Provide in the abstract an informative and balanced summary of what was done and what was found | | 1 | | |  | |
| Introduction | | | |  | | | | |  |
| Background/rationale | | 2 | Explain the scientific background and rationale for the investigation being reported | | 1-4 | | |  | |
| Objectives | | 3 | State specific objectives, including any prespecified hypotheses | | 4 | | | Using data from the 2022 cross-sectional online survey "Sexual Health and HIV/STI in Trans and Non-Binary Communities" (TASG) [19], we investigated the frequency of self-reported HepB diagnoses, self-reported HepB vaccination history, and knowledge of viral hepatitis facts in TNBP in Germany. We also explored potential predictors of self-reported positive HepB vaccination history among those potentially recommended for HepB vaccination to identify prevention opportunities and unmet needs in this population. | |
| Methods | | | |  | | | | |  |
| Study design | | 4 | Present key elements of study design early in the paper | | 4 | | | The TASG study was a participatory cross-sectional study and collaboratively planned, developed, and implemented with representatives of trans and non-binary communities. | |
| Setting | | 5 | Describe the setting, locations, and relevant dates, including periods of recruitment, exposure, follow-up, and data collection | | 4 | | | Between March 1st and July 1st 2022, self-identified TNBP aged 18 or older who reside in Germany were invited to participate in an online survey. Access to the online survey was facilitated through various means, including physical distribution (such as cards featuring a QR code leading to the survey website) and electronic dissemination of the survey link through email lists, social media channels, and online banners on websites. | |
| Participants | | 6 | (*a*) *Cohort study*—Give the eligibility criteria, and the sources and methods of selection of participants. Describe methods of follow-up  *Case-control study*—Give the eligibility criteria, and the sources and methods of case ascertainment and control selection. Give the rationale for the choice of cases and controls  *Cross-sectional study*—Give the eligibility criteria, and the sources and methods of selection of participants | | 4 | | | Between March 1st and July 1st 2022, self-identified TNBP aged 18 or older who reside in Germany were invited to participate in an online survey. | |
|  |  |  | (*b*) *Cohort study*—For matched studies, give matching criteria and number of exposed and unexposed  *Case-control study*—For matched studies, give matching criteria and the number of controls per case | |  | | |  | |
| Variables | | 7 | Clearly define all outcomes, exposures, predictors, potential confounders, and effect modifiers. Give diagnostic criteria, if applicable | | 5-7 | | | All analysed variables and relevant recoding were described within the method section. | |
| Data sources/ measurement | | 8* | For each variable of interest, give sources of data and details of methods of assessment (measurement). Describe comparability of assessment methods if there is more than one group | | 4-7 | | | *See method section.* | |
| Bias | | 9 | Describe any efforts to address potential sources of bias | | 8 | | | We ran a sensitivity analysis to assess the impact of excluding the answer “I don’t know” on the overall proportion of participants with a self-reported positive vaccination history. In a second sensitivity analysis we analysed whether demographic characteristics differed between individuals potentially recommended for HepB vaccination and those included in the bootstrapped stepwise selection procedure. Differences were identified visually with a benchmark of 10% deviation indicating a difference in characteristics. In a third sensitivity analysis, when potential predictors had more than two levels, we dichotomized the variable for each level with a constant base level to identify whether any level stood out as a potential predictor. | |
| Study size | | 10 | Explain how the study size was arrived at | |  | | | Not applicable | |
| Quantitative variables | 11 | Explain how quantitative variables were handled in the analyses. If applicable, describe which groupings were chosen and why | | 7 | | | Descriptive statistics of the participant characteristics include an overview of all main outcomes (self-reported HepB diagnosis, knowledge of hepatitis facts, self-reported HepB vaccination history) and other selected variables. For categorical variables, presentation comprises the total number of respondents and corresponding percentages, with missing values excluded from calculations. For the knowledge variables, we calculated the median number of facts known with the respective interquartile range (IQR) and the proportion of individuals who knew either none, or all four facts. We stratified by self-reported vaccination history (positive vs. negative) for all participants and those potentially recommended for vaccination. | |  |
| Statistical methods | 12 | (*a*) Describe all statistical methods, including those used to control for confounding | | 7-8 | | | We present prevalence ratios and the respective 95% confidence intervals (CI) to allow for comparison across characteristics. Prevalence ratios and CI were estimated by univariate log-binomial regression [23]. We used the bootstrap stepwise selection procedure of Sauerbrei and Schumacher [24] on all variables included in this study to identify potential predictor variables for a positive vaccination history among participants potentially recommended for HepB vaccination. We performed 500 bootstrap replications of the original data set. In each replication, potential predictors were selected by a stepwise backward algorithm that retained only those variables in the logistic regression model that did not exceed our a priori selected cut-off p-value of 0.10. The absolute and relative frequency of inclusion was then used as a criterion for the predictive power of each variable. We considered variables as potential predictors if they were selected in the model with a relative selection frequency (RSF) of >80%. | |  |
|  |  | (*b*) Describe any methods used to examine subgroups and interactions | | 7-8 | | | See above | |  |
|  |  | (*c*) Explain how missing data were addressed | | 7 | | | For categorical variables, presentation comprises the total number of respondents and corresponding percentages, with missing values excluded from calculations. | |  |
|  |  | (*d*) *Cohort study*—If applicable, explain how loss to follow-up was addressed  *Case-control study*—If applicable, explain how matching of cases and controls was addressed  *Cross-sectional study*—If applicable, describe analytical methods taking account of sampling strategy | |  | | | Not applicable | |  |
|  |  | (*e*) Describe any sensitivity analyses | | 8 | | | We ran a sensitivity analysis to assess the impact of excluding the answer “I don’t know” on the overall proportion of participants with a self-reported positive vaccination history. In a second sensitivity analysis we analysed whether demographic characteristics differed between individuals potentially recommended for HepB vaccination and those included in the bootstrapped stepwise selection procedure. Differences were identified visually with a benchmark of 10% deviation indicating a difference in characteristics. In a third sensitivity analysis, when potential predictors had more than two levels, we dichotomized the variable for each level with a constant base level to identify whether any level stood out as a potential predictor. | |  |
| Results | | | | | | | | |  |
| Participants | 13* | (a) Report numbers of individuals at each stage of study—eg numbers potentially eligible, examined for eligibility, confirmed eligible, included in the study, completing follow-up, and analysed | | 9 | | | See **Figure 1.** Study participants inclusion flow chart, TASG 2022. Adapted from Pöge K, Spurgat C, Hamm J, et al. [19] | |  |
|  |  | (b) Give reasons for non-participation at each stage | | 9 | | | See above | |  |
|  |  | (c) Consider use of a flow diagram | | 9 | | | See above | |  |
| Descriptive data | 14* | (a) Give characteristics of study participants (eg demographic, clinical, social) and information on exposures and potential confounders | | 9-10 | | | See **Table 1.** Characteristics of participants with no missing responses regarding the hepatitis B outcomes (n=2,358), Germany 2022. | |  |
|  |  | (b) Indicate number of participants with missing data for each variable of interest | | 9-10 | | | See above | |  |
|  |  | (c) *Cohort study*—Summarise follow-up time (eg, average and total amount) | |  | | |  | |  |
| Outcome data | 15* | *Cohort study*—Report numbers of outcome events or summary measures over time | |  | | |  | |  |
|  |  | *Case-control study—*Report numbers in each exposure category, or summary measures of exposure | |  | | |  | |  |
|  |  | *Cross-sectional study—*Report numbers of outcome events or summary measures | | 10-12 | | | Only 13 of 2,358 participants (0.6%) reported a previous diagnosis of HepB.  The median number of hepatitis facts participants knew was 3 (IQR: 2-4). Overall, 61 (2.8%) of the respondents who answered the questions regarding the hepatitis facts (n=2,203) did not know of any of the four facts, and 795 (36.1%) knew of all of the four facts (Table 1).  56.3% indicated a positive HepB vaccination history, with comparable distribution across age groups. Regarding gender identity, the proportion of self-reported vaccination history varied across gender identities with the biggest difference between those identifying on the male spectrum and those identifying on the female spectrum (PR: 1.29, 95% CI: 1.14, 1.47). | |  |
| Main results | 16 | (*a*) Give unadjusted estimates and, if applicable, confounder-adjusted estimates and their precision (eg, 95% confidence interval). Make clear which confounders were adjusted for and why they were included | | 10-14 | | | No adjusted analysis was conducted.  Crude estimates are presented in **Table 2** and **Table 3**. | |  |
|  |  | (*b*) Report category boundaries when continuous variables were categorized | | 10-14 | | | When applicable boundaries are reported. | |  |
|  |  | (*c*) If relevant, consider translating estimates of relative risk into absolute risk for a meaningful time period | |  | | | Not applicable | |  |
| Other analyses | 17 | Report other analyses done—eg analyses of subgroups and interactions, and sensitivity analyses | | 13-14 | | Additional analyses are presented within the text, **Table 4** and **Figure 2** | | |  |
| Discussion | | | | | | | | |  |
| Key results | 18 | Summarise key results with reference to study objectives | | 15 | | In this manuscript we describe the prevalence of self-reported previous HepB diagnoses and the proportion of self-reported positive vaccination history among TNBP in Germany. In the overall study sample self-reported HepB diagnoses were rare (0.6%) and the self-reported positive HepB vaccination history low (56.3%). Among participants potentially recommended for HepB vaccination, the proportion of positive HepB vaccination history was also low (61.3%) and efforts to increase vaccination uptake are needed. Knowledge of hepatitis facts was identified as a potential predictor of positive vaccination history. | | |  |
| Limitations | 19 | Discuss limitations of the study, taking into account sources of potential bias or imprecision. Discuss both direction and magnitude of any potential bias | | 19-20 | | Some limitations should be considered while interpreting the results. The methodological reliance on self-reporting may introduce recall bias (especially for vaccination history), and the cross-sectional design of the study limits our ability to establish causal relationships. Evidence from Switzerland suggests that self-reported indicators as vaccination history likely overestimate vaccination coverage but perform well in detecting individuals with previous vaccination [33]. Assessing the self-reported vaccination history therefore still gives valuable insights. Other relevant variables that need to be considered regarding HepB vaccination include profession, travel history and migration history that are covered by the standing vaccination recommendations. Thus, the proportion of participants recommended for HepB vaccination in our study might be an underestimate. The unmet needs of these people might differ from the described participants. The reliance on a convenience sample may have introduced a selection bias as more vulnerable individuals that are marginalised in many ways (part of multiple marginalised groups at the same time) might not have been reached. The categorisation of eligibility for the HepB vaccination was based on available responses. This categorisation therefore does not consider any further contextual factors, explicitly that having multiple HepB-vaccinated sexual partners, does not necessarily reflect a higher risk for the disease. We did not include laboratory methods to confirm previous or recent infections nor serology in this study, but this could be considered in future research proposals (e.g. seroprevalence studies). While our findings seem to align with serological national estimates, the prevalence is likely underestimated within our sample due to high proportions of asymptomatic HepB infections. With the data and the relatively small number of participants recommended for vaccination, we could not identify further factors or barriers that potentially affect the vaccine uptake. As mentioned in the method section, we encountered a high number of trans-hostile commentators and calls for fake participation during the recruiting phase, aimed at manipulating the study. This resulted in immense data cleaning, excluding a high number of responses from the dataset. Nevertheless, it is still possible that some of the hostile commentors remained in the dataset while eligible participants were excluded. | | |  |
| Interpretation | 20 | Give a cautious overall interpretation of results considering objectives, limitations, multiplicity of analyses, results from similar studies, and other relevant evidence | | 15-21 | | See discussion and conclusion section. | | |  |
| Generalisability | 21 | Discuss the generalisability (external validity) of the study results | | 19-20 | | See section on limitations. | | |  |
| Other information | |  | | | | | | |  |
| Funding | 22 | Give the source of funding and the role of the funders for the present study and, if applicable, for the original study on which the present article is based | | 22 | | The TASG-study was financed by the German Federal Ministry of Health (BMG) on the basis of a resolution of the German Bundestag. | | |  |

*Give information separately for cases and controls in case-control studies and, if applicable, for exposed and unexposed groups in cohort and cross-sectional studies.

**Note:** An Explanation and Elaboration article discusses each checklist item and gives methodological background and published examples of transparent reporting. The STROBE checklist is best used in conjunction with this article (freely available on the Web sites of PLoS Medicine at http://www.plosmedicine.org/, Annals of Internal Medicine at http://www.annals.org/, and Epidemiology at http://www.epidem.com/). Information on the STROBE Initiative is available at www.strobe-statement.org.
